# Microfiltration of Ovine and Bovine Milk: Effect on Microbial Counts and Biochemical Characteristics

**DOI:** 10.3390/foods9030284

**Published:** 2020-03-04

**Authors:** George Panopoulos, Golfo Moatsou, Chrysanthi Psychogyiopoulou, Ekaterini Moschopoulou

**Affiliations:** Laboratory of Dairy Research, Department of Food Science and Human Nutrition, Agricultural University of Athens, Iera Odos 75, 118 55 Athens, Greece

**Keywords:** microfiltration, ovine milk, bovine milk, casein fractions, alkaline phosphatase, cathepsin D, milk renneting properties

## Abstract

The aim of this research work was to assess the effect of the microfiltration (ceramic membranes 1.4 μm, 50 °C) of partially defatted ovine milk (fat 0.4%) and bovine milk (fat 0.3%) characteristics. Feed milks, permeates and retentates were analyzed for microbial counts, gross composition, protein fractions, the indigenous enzymes cathepsin D and alkaline phosphatase and the behavior during renneting. It was showed that the microbial quality of both ovine and bovine permeate was improved by reduction of the total mesophilic microflora about 4 Log and 2 Log, respectively. The protein contents and the total solids contents of both permeates were significantly (*p* < 0.05) reduced. A further analysis of protein fractions by Reversed Phase -High Performance Liquid Chromatography (RP-HPLC) revealed lower αs_1_- and β-casein and higher κ-casein contents in permeates. The activity of alkaline phosphatase followed the allocation of the fat content, while activity of cathepsin D in permeates was not influenced, although somatic cells counts were removed. Regarding cheesemaking properties, the firmness of ovine curd made from the feed milk did not differ significantly from that made from the permeate. The obtained results suggested that microfiltration could be used for pre-treating of ovine milk prior to cheesemaking.

## 1. Introduction

The membrane process is widely used in the dairy industry for separation or fractionation purposes, depending on the membrane pore size and the applied pressure [1,2]. Microfiltration (MF) involves membranes with a pore size of 0.1–10 μm and operates at pressures of 0.1–8 bars [3,4]. MF is mainly applied to reduce bacteria, spores and somatic cells [1,4,5,6,7] in fluid dairy products extending, in this way, their shelf life [8,9]. Therefore, MF combined with pasteurization is an excellent process to produce Extended Shelf Life (ESL) milk that is considered ‘purer’ and more ’natural’ than standard heat-treated milk. Moreover, MF can be used to fractionate globular milk fat [10], to fractionate and concentrate casein or to purify β-Lactoglobulin (β-Lg) [5,11], while in combination with ultrafiltration, it is used in whey processing. In addition, MF has been studied in regards to pre-treatment of milk for cheese production [12]. All above applications, which are focused on bovine milk, require membranes of different pore sizes. For bacteria reduction, the most used membranes are those of pore size 1.4 μm, since it has been shown that MF of bovine milk through a membrane of 1.4 μm pore size effectively reduces the microbial populations without significantly affecting the major milk components [13,14]. 

Raw ovine milk is richer in total solids and contains higher microbial counts than bovine milk. According to European Community regulation, the microbial criteria for ovine milk define that the two-months rolling geometric average of microbes (with at least two samples per month) grown on Plate Count Agar at 30 °C should be a maximum 1,500,000 per mL. [15]. To the best of our knowledge, studies for MF of ovine milk are rare and concern only the impact on microflora [16,17]. Moreover, there is lack of information about the influence of MF on indigenous enzymes or on the renneting behavior of either ovine or bovine milk. Therefore, the aim of this study is to evaluate the effect of MF, using a ceramic membrane of 1.4 μm pore size, on the composition, microflora, cathepsin D and alkaline phosphatase as well as on cheesemaking properties of ovine and bovine milk. 

## 2. Materials and Methods 

### 2.1. Microfiltration 

**Figure 1:**
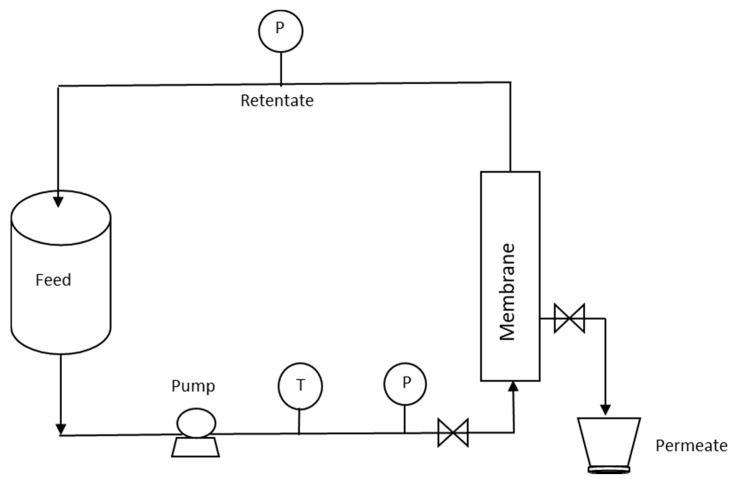
Simplified diagram showing the crossflow microfiltration process.

Raw ovine and bovine milk obtained from the farm of Agricultural University of Athens were defatted to fat content 0.30% and 0.40%, respectively, using a laboratory milk fat separator, and were subsequently heated at 50 °C. Crossflow MF processing (Figure 1) took place on a pilot microfiltration module (PALL Italia s.r.l, MILANO, Italy) under a constant transmembrane pressure (TMP) of 1.5 bar at 50 °C using ceramic membrane P19–40 (Membralox^®^) of 1.4 μm pore size, with length 1020 mm and area 0.24 m^2^. Filtration duration was 15 min with constant flux at 105 ± 32 L·m^−2^·h^−1^ for ovine milk and 314 ± 32 L·m^−2^·h^−1^ for bovine milk. Retention flow rates were 363.23 ± 86.32 L·m^−2^·h^−1^ and 795.83 ± 18.37 L·m^−2^·h^−1^ for bovine and ovine milk retentates, respectively. After processing, the microfiltration unit was cleaned by rinsing in series with water, NaOH 1% solution, water, HNO_3_ 1% solution and water to reach pH 7.0 [17]. Five and three experimental trials were carried out for ovine milk and bovine milk, respectively. Samples codes were as follows: feed ovine milk (O), permeate ovine milk (OP), retentate ovine milk (OR), feed bovine milk (B), permeate bovine milk (BP) and retentate bovine milk (BR). 

### 2.2. Physicochemical Analyses

The pH was measured on a pH meter and acidity was determined by the titration method using 0.11 N NaOH solution. Fat, protein, lactose and total solids contents were determined by means of infrared spectroscopy (Milkoscan FT6000, Foss, Hillerod, Denmark). Ash content was determined by the AOAC method [18]. Total nitrogen (TN) as well as water soluble nitrogen (WSN) were determined by the Kjeldahl method. Phosphorus content was determined by molecular absorption spectrometry [19]. Moreover, calcium content of ovine milk was measured by Atomic Absorption Spectrometry [20] on a Shimadzu AA-6800 Atomic Absorption Spectrophotometer (Shimadzu, Kyoto, Japan) equipped with the autosampler Shimadzu ASC-6100 and the software WizAArd v. 2.30. 

Somatic cell counts (SCC) were determined on Fossomatic (Foss, Hillerod, Denmark). All analyses were performed in duplicate. 

### 2.3. Protein Composition

Casein fractions and whey proteins were determined by the Reversed Phase -High Performance Liquid Chromatography (RP-HPLC) method [21] on a HPLC system consisting of a pump capable of mixing four solvents (Waters 600E, Waters, Milford, MA, USA), a photodiode array detector (Waters 996), a helium degasser, a Rheodyne 7125 injector (Rheodyne Inc., Cotati, CA, USA) and Millennium software (v.3.05.01, Waters, Milford, MA, USA).

### 2.4. Microbial Analyses 

The populations of different microbial groups were estimated by the pour plate method using selective growth media and incubation conditions as follows: total mesophilic microflora on Plate Count Agar at 30 °C for 72 h; coliforms on Violet Red Bile Agar at 37 °C for 24 h; thermophilic lactococci on M17 Agar at 42 °C for 48 h; thermophilic lactobacilli on MRS Agar at 42 °C for 72 h; yeasts and molds on Yeast Glucose Chloramphenicol Agar at 25 °C for 72 h; aerobic spore forming microorganisms on Soluble Nutrition Agar at 42 °C for 48 h; anaerobic spore forming microorganisms on Reinforced Clostridia Medium at 42 °C for 14 days anaerobically. 

### 2.5. Indigenous Enzymes Activity

#### 2.5.1. Cathepsin D Activity 

Cathepsin D activity was determined by the HPLC method proposed by O’Drissol et al. [22] and modified by Hurley et al. [23] using the synthetic peptide Pro-Thr—Glu-Phe-[p-nitro-Phe]-Arg-Leu (Bachem, Switzerland) as substrate. The Waters HPLC system described previously was used. Sample preparation, HPLC conditions and results interpretation were according to Moatsou et al. [24]. 

#### 2.5.2. Alkaline Phosphatase Activity

Alkaline Phosphatase (ALP) activity was determined according to the spectrophotometric method of the International Dairy Federation Standard [25]. The milk sample was diluted with a buffer, pH 10.6, containing disodium phenyl phosphate (substrate) and was incubated at 37 °C for 1 h. After incubation, any active ALP had liberated phenol from the substrate. The phenol was detected by adding 2,6-dibromoquinonechlorimide to detect blue color (dibromoindophenol), which was measured on a spectrophotometer (model Lambda 20, Perkin Elmer, Waltham, MA, USA) at 610 nm. The ALP activity was expressed in μg of phenol per mL of milk. 

### 2.6. Rennet Clotting Behavior 

The behavior of MF permeates and retentates during clotting with rennet (Nature extra 1125; Hansen Denmark) and was determined on a Formagraph (Lattoninamograph; Foss, Padova, Italia). Milk clotting time and curd firmness after 30 min were recorded. 

### 2.7. Statistical Analysis

The obtained data were subjected to statistical analysis using the software Statgraphics (Statistical Graphics Corp. Rockville, Maryland, MD, USA). Comparisons of means were made using the Least Significant Difference test (LSD, *p* < 0.05). 

## 3. Results and Discussion

### 3.1. Physicochemical Composition 

The gross composition, pH value and acidity of both ovine and bovine milk are shown in Table 1. First, as feed milks were not skim, i.e., 0.05% fat, a significant (*p* < 0.05) reduction of fat contents of permeates was observed. This was expected since MF, even through a membrane of 1.4 μm pore diameter, removes fat [4]. Second, protein content was significantly (*p* < 0.05) lower in both ovine and bovine permeate than in the respective feed milks O and B. This was probably due to the significant lower casein nitrogen (Table 2). In contrast, the water-soluble nitrogen components did not exhibit a significant difference between permeates and retentates of both milks. The casein nitrogen to total nitrogen (CN/TN) ratio showed 1.3% and 5.1% retention of casein for bovine and ovine permeate, respectively, despite the similar average diameter of casein micelles, i.e., 193 nm in ovine and 180 nm in bovine milk [26]. Because MF operates at relatively low pressures, it is susceptible to fouling and to formation of a secondary layer on the membrane by gelatinous material when higher pressure and fluxes are applied [3]. In addition, the length of the tubular membrane that was used was long enough (1.02 m) for the formation of a secondary layer, since it has been shown that tubular membranes 1.2 m long usually operate under a deposit layer [27]. The flux in this experiment was constant during the 15 min of MF process. Therefore, the retention of casein micelles could be due to the formation of a secondary membrane on membrane surface. Other researchers have reported non-significant casein retention during MF (1.4 μm) of bovine milk [13,28]. 

The total solids content of permeates were similarly affected by the casein retention (Table 1). Casein retention affects also the phosphorus and calcium contents, since part of these inorganic elements are associated with casein micelles. Because of this, the mean phosphorus content of bovine permeate milk was significantly lower than of bovine feed milk and its retentate. The same trend was observed for the mean phosphorus content of ovine permeate milk. Moreover, the mean calcium content of ovine permeate milk was 148.56 ± 7.77 mg/100g, and it was significantly (*p* < 0.05) lower than in feed milk (198.01 ± 15.86 mg/100g) and retentate (191.06 ± 6.94 mg/100g). Ash contents were like phosphorus and calcium contents, i.e., significantly lower in ovine and bovine permeates than in the respective feed milk (Table 1). 

Finally, pH values were not affected, but acidities of both OP and BP were significantly (*p* < 0.05) lower than in feed milks. Milk acidity is related to protein content and, thus, the lower acidity values of permeates could be associated with the lower protein content that both permeates presented. 

### 3.2. Protein Composition 

Further analysis of proteins by RP-HPLC revealed the results shown in Table 3. It is obvious that the application of crossflow MF, even with a membrane of 1.4 μm pore size, resulted in the fractionation of casein micelles, since protein permeation depends on the interaction between membrane pore and protein size. Both ovine and bovine milk permeates had significantly (*p* < 0.05) higher κ-casein (κ-CN) contents and lower β-casein (β-CN) contents than their counterpart’s retentates. Similar, but not statistically significant, results have been reported by Tziloula et al. [28], who showed that micelles of bovine milk with a diameter greater than 550 nm were retained during MF (1.4μm). In ovine permeate, except for β-CN, the α_s1_-CN and α_s2_-CN contents were significantly (*p* < 0.05) lower. The loss of casein fractions and especially those of κ-CN in retentate may be due to the damage of the casein micelles surface, which is caused by the shear forces in the membrane circuit pump [11]. In the case of ovine milk, the shear forces were lower because of the lower flux compared to bovine milk and, thus, more κ-CN remained in permeate. On the other hand, α-lactalbumin (α-La) and β-Lg were concentrated in the permeates because of their smaller volume. The particle diameter of bovine α-La and β-Lg was less than 10 nm [29]. 

### 3.3. Somatic Cell Counts

Somatic cells were completely removed from both ovine and bovine permeates, and were concentrated in retentates (Table 1). One of the main purposes of applying MF is the 100% reduction in SCC. In the case of skimmed milk, this can be achieved using a MF membrane of 1.4 μm pore size [4,30], whereas, for raw whole bovine milk, membranes of an average pore size from 12 μm to 5 μm with permeate fluxes between 2000 L·m^−2^·h^−1^ and 1460 L·m^−2^·h^−1^ are needed to remove 93–100% of the SCC [5]. 

### 3.4. Microbial Counts 

**Figure 2:**
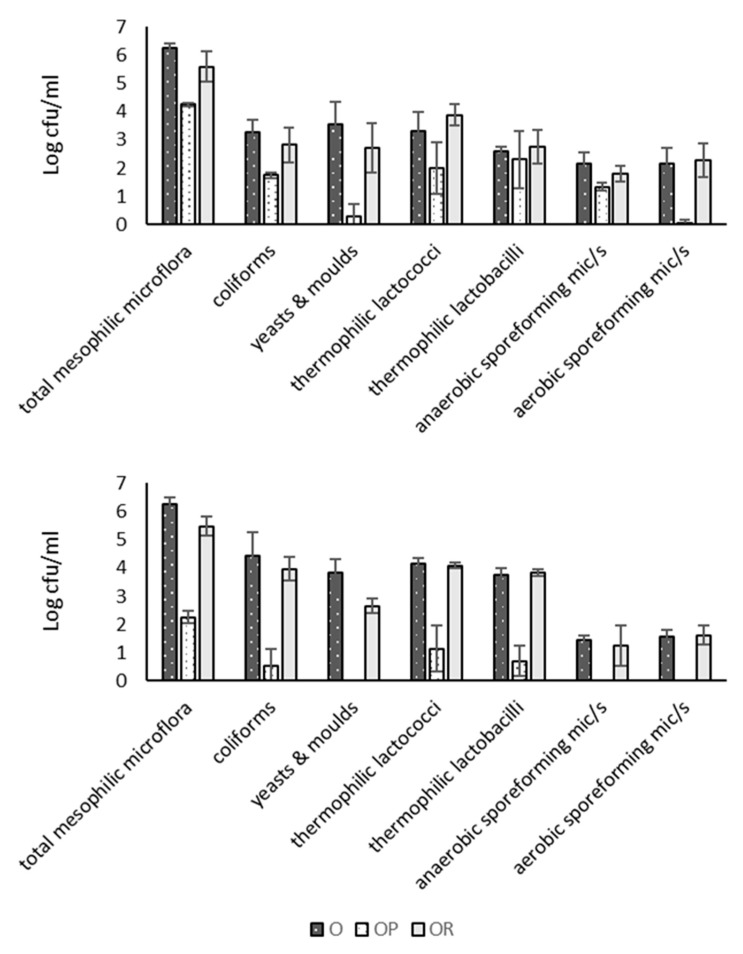
Effect of microfiltration on microbial counts (Log cfu.mL^−1^) of partially defatted bovine (B) or ovine (O) milk, bovine permeate (BP), bovine retentate (BR) (mean ± SD, n = 3), ovine permeate (OP) and ovine retentate (OR) (mean ± SD, n = 5).

The populations of microorganisms are presented in Figure 2. It is obvious that MF affected the microbial groups in a different way, since the retention of specific bacteria depends on their cellular volume [31,32] and on their initials counts in the feed milk. In bovine permeate, total mesophilic microorganisms, coliforms, thermophilic lactococci and lactobacilli were reduced from 1.5 to 2.5 Log, while in ovine permeate, the microbial counts reduction was more efficient, e.g., total mesophilic microflora was reduced about 4 Log. Similar reduction of bacterial load has been reported for bovine permeate [13,17,31], while a reduction 2–3 Log has been reported for ovine permeate obtained with flux 200 L·m^−2^·h^−1^ under pressure 0.6 bar at 40 °C [17]. It has been shown that by using a membrane of 1.4 μm pore size and fluxes of over 640 L·m^−2^·h^−1^, 99.7% of the bacteria can be removed from skim bovine milk [33]. Moreover, by cold MF (1.4 μm) at 6 °C, a method to inhibit bacteria growth in the system, an average of 3.4 Log reduction in vegetative bacteria can be achieved [9]. In the present study, the MF system and the applied conditions should meet the nominal reduction of bacteria counts 3–4 Log at flux 166 L·m^−2^·h^−1^. In the case of ovine milk, the flux was 105 ± 32 L·m^−2^·h^−1^ and hence, this milk kind in combination with its higher protein content, which probably caused a thicker deposit layer on the membrane, retained more microorganisms than bovine milk. 

Regarding sporeforming microorganisms, they were completely removed from ovine permeate, while in bovine permeate, they were reduced about 0.5–2 Log. The total retention of such microorganisms in the case of ovine milk was also attributed to a thicker protein layer formation on the membrane. In contrast, the insufficient retention of them in bovine retentate followed the trend of all other microbial groups and was attributed to the MF applied conditions in combination with the kinds of the present microorganisms. For example, *Lactobacillus casei*, a thermophilic microorganism, has cell width size 0.5–0.8 μm, whereas *B. cereus*, an aerobic sporeforming microorganism, is 1 μm [32]. Griep et al. [7], using cold MF (1.4 μm) for skim bovine milk, showed that *B. licheniformis* spores were reduced 2.17 log, while *Geobacillus sp*. spores were completely removed. 

### 3.5. Alkaline Phosphatase Activity 

The activity of Alkaline Phosphatase (ALP), an indigenous milk enzyme with technological importance in respect to the heat treatment of milk, was significantly reduced about 50–59% in bovine and ovine MF permeates (Table 4). This was because the γ-type of ALP is mostly found on the milk fat globule membrane [34] and, thus, its activity followed the partition of the milk fat after microfiltration. The ALP activity of permeate bovine was found to be as high as 407 μg phenol/mL, and it was similar to the value 445 μg phenol/mL reported for bovine skim milk, i.e., 0.05% fat [35]. The same researchers have reported the ALP of ovine skim milk as 4382 μg phenol/mL. 

### 3.6. Cathepsin D Activity 

**Figure 3:**
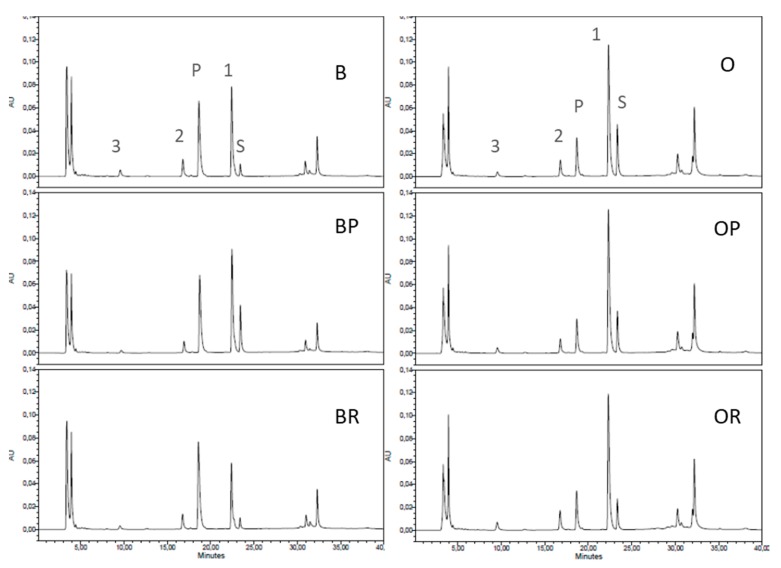
Proteinase activities in the acid whey fractions of feed bovine milk (B), bovine permeate (BP) and bovine retentate (BR), or feed ovine milk (O), ovine permeate (OP) and ovine retentate (OR), after incubation with the substrate Pro-Thr-Glu-Phe-[p-nitro-Phe]-Arg-Leu at pH 3.2 for 12 h at 37 °C. S: residual substrate; P: cathepsin D-like product; peaks 1, 2 and 3: other proteinases.

Cathepsin D, a lysosomal aspartic proteinase, is found in somatic cells and acts on κ-, α_s1_- and β-casein similarly to how it acts on chymosin [36]. The assay used for its determination was based on the presence of peak P (Figure 3), which is the product that resulted from the action of cathepsin D on the substrate. The quantitative determination (Table 4) was based on the chromatographic area of this peak. Peak 1 is another aspartic proteinase, whereas peaks 2 and 3 are possible cysteine- proteinases [24]. Cathepsin D was determined in the acid whey fraction of the feed milks, MF permeates and retentates, and there was no significant difference among them, since whey is easily filtered through a membrane of 1.4 μm pore size. However, it is noteworthy that although somatic cells were completely removed from permeates, the acid proteinases associated with them were present. It seems that the shear forces induced by circulation removes cathepsin from somatic cells and, thus, it remains in the permeate. The higher flux in the case of bovine milk might cause higher dissociation from somatic cells and, therefore, the product resulted from cathepsin activity was higher than that of ovine milk.

### 3.7. Renneting Behavior

Regarding the renneting behavior, there was not a significant difference among the feed, the permeate and retentate for both milks as far as the milk clotting time was concerned (Table 5). Milk clotting time depends mainly on the ratio enzyme/substrate, milk pH, temperature and calcium ions. Although in the case of ovine milk, calcium and phosphorous contents in the permeate were significantly lower than in feed milk or MF retentate, and the clotting time was not significantly affected. In addition, the firmness of the curd made from ovine permeate did not differ significantly from the curd made from ovine feed milk, although the casein content of milk has a significant influence on its maximum firmness [26]. In contrast, the firmness of curd made from bovine permeate was significantly lower than the curd made from the feed milk. 

## 4. Conclusions

From the obtained results it appears that the application of crossflow MF using a membrane of 1.4 μm pore size, at 50 °C and TMP 1.5 bar, influences the ovine milk (0.4% fat) in a similar way to the bovine milk (0.3% fat). More specifically, the applied crossflow MF improved the microbial quality, but significantly reduced (*p* < 0.05) the protein content and, consequently, the total solids content of both the ovine and bovine permeate. In addition, crossflow MF influenced the distribution of casein fractions between permeate and retentate with αs1- and β-CN retention being more pronounced in the case of ovine milk. The activity of indigenous enzyme ALP followed the allocation of the fat content, while the activity of cathepsin D was not influenced. Regarding cheesemaking properties, the firmness of the curd made from the ovine feed milk did not differ from the curd made from the ovine permeate. It is concluded that crossflow MF under the studied conditions can be used as pre-treatment to improve the microbial quality of ovine milk prior to cheesemaking. However, further study is needed to optimize the conditions of crossflow MF processing for this kind of milk. 

## Figures and Tables

**Table 1:** Effect of microfiltration on pH, acidity, somatic cells counts (SCC) and chemical composition (%) of partially defatted bovine (B) or ovine (O) milk, bovine permeate (BP), bovine retentate (BR) (mean ± SD, n = 3), ovine permeate (OP) and ovine retentate (OR) (mean ± SD, n = 5).

	Bovine			Ovine		
	B	BP	BR	O	OP	OR
**pH**	6.68 ± 0.02	6.67 ± 0.03	6.68 ± 0.02	6.60 ± 0.07	6.53 ± 0.07	6.49 ± 0.08
**Acidity**	0.15 ± 0.02 ^a,^*	0.13 ± 0.00 ^b^	0.14 ± 0.01 ^a,b^	0.22 ± 0.02 ^a^	0.19 ± 0.02 ^b^	0.22 ± 0.02 ^a^
**Fat**	0.29 ± 0.10 ^a,b^	0.05 ± 0.02 ^a^	0.46 ± 0.19 ^b^	0.41 ± 0.09 ^a^	0.16 ± 0.01 ^b^	0.43 ± 0.11 ^a^
**Protein**	3.43 ± 0.15 ^a^	3.03 ± 0.21 ^b^	3.63 ± 0.15 ^a^	5.71 ± 0.28 ^a^	4.68 ± 0.17 ^b^	5.78 ± 0.28 ^a^
**Lactose**	4.91 ± 0.12	4.96 ± 0.23	4.96 ± 0.22	4.83 ± 0.05	4.76 ± 0.04	4.80 ± 0.05
**Total Solids**	8.88 ± 0.29 ^a,b^	8.3 ± 0.21 ^a^	9.24 ± 0.44 ^b^	10.87 ± 0.46 ^a^	9.32 ± 0.21 ^b^	10.95 ± 0.46 ^a^
**Ash**	0.78 ± 0.01 ^a^	0.75 ± 0.02 ^b^	0.81 ± 0.02 ^a^	0.94 ± 0.04 ^a^	0.82 ± 0.03 ^b^	0.96 ± 0.04 ^a^
**Phosphorus (mg/100 g)**	105.35 ± 5.73 ^a^	97.54 ± 1.43 ^b^	107.19 ± 1.65 ^a^	149.62 ± 5.46 ^a^	127.85 ± 5.50 ^b^	154.70 ± 8.36
**SCC**	417500.00 ± 43100.00 ^a^	0	653000.00 ± 73500.00 ^b^	660400.00 ± 199600.00 ^a^	0	570600.00 ± 159000.00 ^b^

* For the same milk, means with different superscript in the same row differ significantly (*p* < 0.05).

**Table 2:** Effect of microfiltration on the nitrogen components (%) of partially defatted bovine (B) or ovine (O) milk, bovine permeate (BP), bovine retentate (BR) (mean ± SD, n = 3), ovine permeate (OP) and ovine retentate (OR) (mean ± SD, n = 5).

	Bovine			Ovine		
	B	BP	BR	O	OP	OR
Total nitrogen (TN)	0.55 ± 0.03 ^a^	0.48 ± 0.02 ^b^	0.55 ± 0.02 ^a^	0.88 ± 0.04 ^a^	0.71 ± 0.02 ^b^	0.90 ± 0.03 ^a^
Casein nitrogen (CN)	0.43 ± 0.04 ^a^	0.35 ± 0.02 ^b^	0.43 ± 0.03 ^a^	0.67 ± 0.03 ^a^	0.51 ± 0.04 ^b^	0.68 ± 0.03 ^a^
Water soluble nitrogen	0.12 ± 0.01	0.13 ± 0.01	0.13 ± 0.02	0.21 ± 0.04	0.20 ± 0.03	0.22 ± 0.04
CN/TN	0.75 ± 0.01	0.74 ± 0.04	0.75 ± 0.07	0.78 ± 0.01 ^a^	0.74 ± 0.02 ^a^	0.78 ± 0.0 ^b^

For the same milk, means with different superscript in the same row differ significantly (*p <* 0.05).

**Table 3:** Effect of microfiltration on casein fractions (% of total protein) and the main whey proteins (% of total protein) of partially defatted bovine (B) or ovine (O) milk, bovine permeate (BP), bovine retentate (BR) (mean ± SD, n = 3), ovine permeate (OP) and ovine retentate (OR) (mean ± SD, n = 5).

	Bovine			Ovine		
	B	BP	BR	O	OP	OR
κ-CN	11.02 ± 0.30 ^a^	11.47 ± 0.48 ^b^	11.56 ± 0.24 ^b^	9.71 ± 0.43 ^a^	10.14 ± 0.51 ^b^	9.77 ± 0.17 ^a^
αs1-CN	28.62 ± 0.82	28.19 ± 0.60	28.90 ± 1.26	29.02 ± 0.81 ^a^	27.42 ± 0.53 ^b^	29.41 ± 0.82 ^a^
αs2-CN	8.96 ± 0.77	9.03 ± 0.63	8.93 ± 0.83	12.51 ± 0.95 ^a^	11.66 ± 0.88 ^b^	12.58 ± 0.70 ^a^
β-CN	34.09 ± 0.77 ^a^	32.59 ± 0.58 ^b^	33.11 ± 1.00 ^b^	32.01 ± 1.66 ^a^	30.67 ± 1.03 ^b^	31.83 ± 0.85 ^a^
α-la	2.69 ± 0.28 ^a,b^	2.91 ± 0.33 ^a^	2.52 ± 0.23 ^b^	3.72 ± 0.24 ^a^	4.41 ± 0.28 ^b^	3.80 ± 0.46 ^a^
β-lg	8.07 ± 0.71	8.49 ± 0.91	7.75 ± 0.65	8.11 ± 0.57 ^a^	9.48 ± 0.62 ^b^	7.70 ± 0.29 ^a^

For the same milk, means with different superscript in the same row differ significantly (*p <* 0.05).

**Table 4:** Effect of microfiltration on alkaline phosphatase (ALP) activity (μg phenol/mL) and protease activities (area × 10^6^ of peaks of RP-HPLC) in the whey fraction of partially defatted bovine (B) or ovine (O) milk, bovine permeate (BP), bovine retentate (BR) (mean ± SD, n = 3), ovine permeate (OP) and ovine retentate (OR) (mean ± SD, n = 5).

	Bovine			Ovine		
	B	BP	BR	O	OP	OR
ALP	810 ± 127 ^a^	407 ± 111 ^b^	964 ± 217 ^a^	4728 ± 958 ^a^	2832 ± 757 ^b^	4132 ± 1009 ^a^
Proteases in whey						
Cathepsin D-like product	0.99 ± 0.08 ^a^	0.97 ± 0.08 ^a^	1.24 ± 0.03 ^b^	0.39 ± 0.06	0.34 ± 0.05	0.36 ± 0.01
Peak 1	1.04 ± 0.08 ^a^	1.06 ± 0.14 ^a^	0.64 ± 0.11 ^b^	2.02 ± 0.25	1.93 ± 0.15	1.81 ± 0.18
Peak 2	0.21 ± 0.04	0.19 ± 0.07	0.21 ± 0.04	0.07 ± 0.01 ^a^	0.16 ± 0.02 ^b^	0.19 ± 0.02 ^b^
Peak 3	0.11 ± 0.01 ^a^	0.04 ± 0.01 ^b^	0.04 ± 0.01 ^b^	0.14 ± 0.05	0.13 ± 0.06	0.25 ± 0.03

For the same milk, means with different superscript in the same row differ significantly (*p <* 0.05).

**Table 5:** Effect of microfiltration on milk clotting time (r) and curd firmness (A_30_) of partially defatted bovine (B) or ovine (O) milk, bovine permeate (BP), bovine retentate (BR) (mean ± SD, n = 3), ovine permeate (OP) and ovine retentate (OR) (mean ± SD, n = 5).

	Bovine			Ovine		
	B	BP	BR	O	OP	OR
r (min)	20.71 ± 1.13	22.04 ± 2.41	21.25 ± 2.08	14.15 ± 2.44	14.41 ± 2.25	14.34 ± 2.38
A_30_ (mm)	19.12 ± 2.93 ^a^	12.58 ± 3.22 ^b^	17.81 ± 2.26 ^a,b^	42.71 ± 3.73 ^a^	39.70 ± 4.35 ^a,b^	46.29 ± 4.55 ^b^

For the same milk, means with different superscript in the same row differ significantly (*p* < 0.05).
